# Application of an Integrated and Open Source Workflow for LC-HRMS Plant Metabolomics Studies. Case-Control Study: Metabolic Changes of Maize in Response to *Fusarium verticillioides* Infection

**DOI:** 10.3389/fpls.2020.00664

**Published:** 2020-06-05

**Authors:** Biancamaria Ciasca, Alessandra Lanubile, Adriano Marocco, Michelangelo Pascale, Antonio F. Logrieco, Veronica M. T. Lattanzio

**Affiliations:** ^1^Institute of Sciences of Food Production, National Research Council, Bari, Italy; ^2^Department of Sustainable Crop Production, Università Cattolica del Sacro Cuore, Piacenza, Italy

**Keywords:** metabolomics, *Fusarium verticillioides*, maize, high-resolution mass spectrometry (HRMS), open-source workflow

## Abstract

Liquid chromatography coupled with high-resolution mass spectrometry (LC-HRMS) represents the most powerful metabolomics platform to investigate biological systems. Reproducible and standardized workflows allow obtaining a meaningful biological interpretation. The purpose of this study was to set up and apply an open-source workflow for LC-HRMS plant metabolomics studies. Key steps of the proposed workflow were as follows: (1) experimental design, (2) sample preparation, (3) LC-HRMS analysis, (4) data processing, (5) custom database search, (6) statistical analysis, (7) compound identification, and (8) biochemical interpretation. Its applicability was evaluated through the study of metabolomics changes of two maize recombinant inbred lines with contrasting phenotypes with respect to disease severity after *Fusarium verticillioides* infection of seedlings. Analysis of data from the case-control study revealed abundance change in metabolites belonging to different metabolic pathways, including two amino acids (L-tryptophan and tyrosine), five flavonoids, and three *N*-hydroxynnamic acid amides.

## Introduction

Metabolomics is a powerful approach for comprehensive investigation of metabolite variations in biological systems (Blaženovic et al., 2018). Currently, liquid chromatography coupled with high-resolution mass spectrometry (LC-HRMS) represents the most powerful metabolomics platform. Untargeted metabolite mass profiles can be used for biological interpretations; however, approaches that do not require the identification of the metabolic features should be used with extreme caution, because they may lead to false interpretations. The identification of metabolites with a high level of confidence is required in order to improve the meaning of metabolomics in biological systems, such as plant–pathogen interaction and possible applications. Within this context, having available well-established computational tools and workflows is essential for highly reproducible and repeatable metabolomics studies. In addition, knowledge-based workflows for metabolite annotations are highly desirable to complement information relevant to mass spectrometry (MS) peaks relationships (adducts and neutral losses), MS/MS data, and retention time modeling with biochemical knowledge. Sharing workflows (research data and processing tools) help to validate the findings reported in publications and, more importantly, let researchers freely reuse the data as they are, or as a reliable basis to move forward. Therefore, a priority issue in the metabolomics field is the validation and harmonization of untargeted approaches. In this regard, a possible approach for validation of untargeted MS methods to be used for food fraud detection has been proposed by Cavanna et al. (2018). The proposed approach is based on tools such as quality control samples to check analytical performances, cross-validation, and receiver operating characteristic curves to assess the goodness of the model, which is its ability to correctly classify one condition and/or outcome from another. Moreover, criteria to validate identified markers are proposed such as survey of blind real samples, analysis of reference samples, and integration of multiplatform data.

*Fusarium verticillioides* (Sacc.) Nirenberg is a predominant endophyte and pathogen of maize causing substantial yield losses and reduction of grain quality. Maize can be infected by the fungus at all growth stages, from the early vegetative phases to maturity. The fungus can be transmitted through infected kernels and causes systemic infection that eventually contribute to the development of seedling diseases (Bacon and Hinton, 1996), including seedling rot, root rot, and stalk rot (Yates et al., 2003; Baldwin et al., 2014). The interest in this fungus has also arisen from mycotoxin accumulation in preharvest infected plants or in stored grains. *Fusarium verticillioides* mycotoxins, including fumonisins, have been associated with chronic or acute mycotoxicoses in livestock (Gelderblom et al., 1988).

Maize defends itself against *F. verticillioides* infection by activating a multicomponent defense response (Lanubile et al., 2017). The molecular basis of resistance to this fungus in maize has been investigated through next-generation sequencing approaches. These tools helped in the dissection of complex traits as resistance to *Fusarium* infection of seedlings (FIS) and mature kernels, and several markers and candidate genes were proposed through the analysis of the transcriptional profiles of resistant and susceptible maize kernels (Campos-Bermudez et al., 2013; Lanubile et al., 2014; Maschietto et al., 2016; Wang et al., 2016) and different maize panels and populations (Ju et al., 2017; Maschietto et al., 2017; Septiani et al., 2019; Stagnati et al., 2019). Additional potential biomarkers for *F. verticillioides* resistance were proposed through the analysis of the proteomic profile of susceptible maize embryos after *F. verticillioides* inoculation (Campo et al., 2004). However, the strong influence of the environment on this trait makes the identification of markers in response to fungal infection difficult, and new approaches are required to fill the gap between the genotype and the observed phenotype.

To complement molecular studies, metabolomics-based technologies provide a powerful tool to identify candidate metabolites involved in resistance mechanisms. The metabolites involved in cereal resistance to *Fusarium* infection derive from primary and secondary plant metabolism and can be roughly classified in six major groups: fatty acids, amino acids and derivatives, carbohydrates, amines and polyamines, terpenoids, and phenylpropanoids (Gauthier et al., 2015; Atanasova-Penichon et al., 2016).

Most of the available metabolomics studies are based on platforms such as GC-MS, nuclear magnetic resonance (NMR), or LC-MS and are focused on resistance-related metabolites in wheat and barley after *Fusarium graminearum* infection (Hamzehzarghani et al., 2005, 2008; Browne and Brindle, 2007; Paranidharan et al., 2008; Bollina et al., 2010; Cajka et al., 2014; Gunnaiah and Kushalappa, 2014; Kage et al., 2017). Few studies have been carried out to investigate the metabolic defense induced by *F. verticillioides* in maize. Targeted analytical approaches have been developed to study oxylipins produced by *F. verticillioides* in maize seedling roots (Ludovici et al., 2014), phenylpropanoids in maize pericarp (Sampietro et al., 2013), and the effect of antioxidants, namely, ferulic acid, tocopherols, and carotenoids, in resistance to *Fusarium* ear rot and fumonisin accumulation in maize (Picot et al., 2013). To the best of our knowledge, the only available studies based on untargeted metabolite profiling have been reported by Campos-Bermudez et al. (2013) and Righetti et al. (2019) aiming at detecting metabolic changes associated to infection of *F. verticillioides* in maize. The first study was based on gas chromatography/MS, which can detect only volatile metabolites (Campos-Bermudez et al., 2013). The second one used LC-HRMS to investigate differences in metabolic profiles among maize commercial hybrids in relation to fumonisin accumulation in naturally contaminated samples under open-field conditions. This study pointed out a significant influence from the hybrid genotype, the environmental growing conditions, and lipid composition on fumonisin accumulation.

The purpose of this work was to set up a workflow for LC-HRMS plant metabolomics studies based on open-source data processing tools, to provide a shareable approach that could be the basis for future validation.

The developed workflow enables processing of data from targeted and untargeted LC-HRMS analysis (profiling and compound annotation). It is based on free and user-friendly software facilitating data reuse and replication, interrogation, and verification of obtained results. The applicability of the developed approach is here demonstrated through a preliminary investigation of the metabolic response of maize induced by *F. verticillioides* infection. Evaluation studies were performed on a case-control study, whereas further validation experiments will be carried out in a future work.

## Materials and Methods

### Materials and Reagents

Acetonitrile, methanol (both HPLC grade), and glacial acetic acid were purchased from VWR International (Milan, Italy), whereas ammonium acetate for MS was purchased from Sigma-Aldrich (Milan, Italy). Ultrapure water (18 MΩ) was produced by a Millipore Milli-Q system (Millipore; Bedford, MA, United States). (^13^C_34_)−FB_1_ internal standard was purchased from BiopureReferenzsubstanzen (Tulln, Austria) as liquid calibrant (25 mg/L) in acetonitrile.

### Plant Material and *in vitro* Assay

The maize recombinant inbred lines (RILs) 5_3, 14_84, and 18_27 with contrasting phenotypes with respect to the disease severity after FIS were obtained from Scuola Superiore Sant’Anna, Pisa, Italy (Dell’Acqua et al., 2015; Septiani et al., 2019).

**FIGURE 1:**
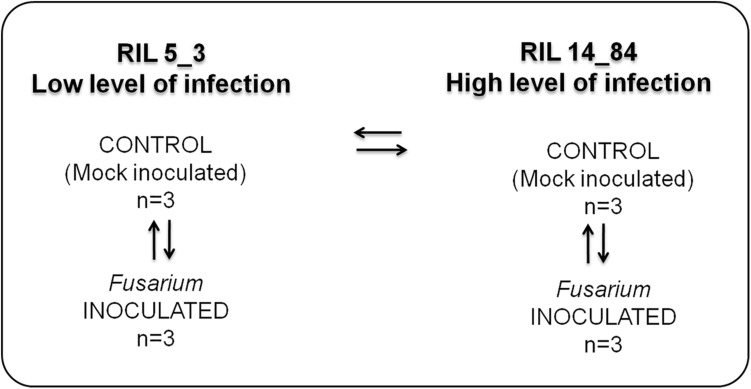
Experimental design applied to study the metabolic responses in the two maize RILs (5_3 and 14_84) with contrasting phenotypes with respect to disease severity after *F. verticillioides* infection of seedlings (FIS). The RILs are labeled as “high” and “low infection” on the basis of phenotypic value of FIS as reported in Figure 3.

Forty seeds with similar size and without visible damages were selected for each RIL, 20 to be used as a treated sample (*Fusarium* inoculation) and 20 to be used as a control (mock inoculation) (Figure 1). Seeds were surface-sterilized in a solution of 70% ethanol shaken for 5 min at 50 rpm to reduce seed-borne contaminations. Ethanol was removed, and seeds were washed by sterilized bidistilled water for 1 min, then by a commercial bleach solution for 10 min, and finally rinsed three times (5 min each) with sterilized bidistilled water.

Six rolled towel assays (RTAs), for control and treated conditions, were prepared for each RIL. Three towels of germination paper (Anchor Paper, St. Paul, MN, United States) for each RTA were moistened with sterilized bidistilled water; 20 seeds were placed evenly spaced on two base towels and were covered with the third towel. In the treated RTA, the 20 seeds were each inoculated by pipetting 100 μL of a 3.5 × 10^6^ mL^–1^ spore suspension of *F. verticillioides* ITEM10027 (MPVP 294). The strain was isolated from maize in South Tuscany, Italy, by the Department of Sustainable Crop Production, Piacenza, Italy, and deposited in their fungal collection and also in the Institute of Sciences and Food Production, National Research Council of Italy, Bari^1^. The towels were then rolled up, placed vertically in a bucket and kept in transparent plastic bags separately for treated and control to avoid cross-contamination. Rolled towel assays were incubated at 25°C in the dark for 7 days.

After incubation, RTAs were laid on a work bench and opened to phenotype seedlings for FIS, seedling length (SL), and seedling weight (SW). All traits were measured on each seedling in the control RTAs, named as FISC, SLC, and SWC, and in the treated RTAs, named as FIST, SLT, and SWT. The FIS was assessed on each seedling by a visual evaluation of seedling size and visible colonization of *F. verticillioides* in a scale from 1 to 5 as previously described (Lanubile et al., 2015; Bernardi et al., 2018; Stagnati et al., 2019). On this scale, 1 corresponds to complete absence of disease symptoms, and five corresponds to complete presence of disease symptoms. Seedling length was determined by measuring the length of the seed from the tip of the shoot to the tip of the root, in centimeters. Seedling weight was determined by measuring the weight of the whole germinated seed using a laboratory scale, in grams.

For FIS, SL, and SW trait analyses, standard deviations of the means were calculated on 20 seedlings of three RTAs. Two-factor analysis of variance (*P* < 0.05) was performed on the observed means of FIS, SL, and SW traits of control and treated seedlings of 5_3 and 14_84 lines. Genotypes and treatment were considered as fixed factors to test their significances and the significance of their interaction.

Kernel tissues were dissected from maize seedling samples of the RILs for the further metabolomics analysis at 7 days after inoculation with *F. verticillioides* and their respective controls (mock inoculation), cryogenically milled, and stored at −80°C until the analysis.

### Step 1: Experimental Design

The experimental design used in this study is shown in Figure 1. It entailed four treatments (two RILs, 5_3 and 14_84; two inoculations with water (mock) or pathogen, respectively). For each treatment, three replicates were performed.

The treatment combinations tested were as follows: control (mock)– versus *Fusarium-*inoculated kernels for both RILs 5_3 and 14_84 to have information on metabolites generated after the fungal infection (responsive metabolites) and then control (mock line 5_3) versus control (mock line 14_84) to obtain information on differences in constitutive metabolites potentially related to the susceptibility. Three biological replicates were included in each group to be statistically confident in the obtained results.

### Step 2: Sample Preparation

Inoculated and control maize kernels were ground in liquid nitrogen with a mortar and pestle and lyophilized. The lyophilized sample (0.5 g) was extracted first with 2 mL methanol, by 30-min shaking (extract A). After centrifugation (15 min, 4,000 × *g*), the extract A was removed, and the residue was extracted again with 2 mL of a mixture acetonitrile/water (84:16) with 1% acetic acid, by 30-min shaking (extract B). The extract B was recovered by centrifugation (15 min, 4,000 × *g*). Extracts A and B were unified and then diluted fourfold with water. An aliquot of 200 μL (6.25 mg of matrix) was fortified by adding 20 μL of isotope-labeled internal standard ([^13^C_34_]–fumonisin B_1_ at 5 ng/μL). Samples were filtered through a 0.22-μm RC syringe filter prior injection into the LC-HRMS apparatus.

### Step 3: LC-HRMS Analysis

Liquid chromatography–HMRS analysis was performed on a Q-Exactive^TM^ Plus mass spectrometer, equipped with a heated electrospray ion source (HESI II) coupled to an Ultimate 3000 UHPLC system (all from Thermo Fisher Scientific, San Jose, CA, United States).

The LC column was a Gemini^®^ C18 column (150 × 2 mm, 5-μm particles) (Phenomenex, Torrance, CA, United States), preceded by a Gemini^®^ C18 guard column (4 × 2 mm). The column oven was set at 40°C. The flow rate of the mobile phase was 200 μL/min, whereas the injection volume was 20 μL. Eluent A was water, and eluent B was methanol, both containing 0.5% acetic acid and 1 mM ammonium acetate. The following gradient was used: the proportion of eluent B was kept constant at 10% for 5 min and then linearly increased to 80% in 36 min. Finally, it was raised to 100% and kept constant for 5 min. The column was re-equilibrated with 10% eluent B for 9 min. The HESI II ion source was operated in positive ion mode and in negative mode, with the following settings: sheath gas: 30 arbitrary units, auxiliary gas: 15 arbitrary units, spray voltage: 3 kV, S-lens RF level: 50 (arbitrary units), capillary temperature: 320°C, heater temperature: 300°C. A divert valve was used, and the eluent was directed to waste from 0 to 4 min and from 41 min until the end of the re-equilibration step. Samples were injected in random order.

High-resolution mass spectrometry chromatograms were acquired in positive and negative ionization mode, respectively (a distinct run for each modality). A total of six scan events were combined: one full scan event (mass range, 100–1,000 *m/z*); resolving power 70,000 full width at half maximum (FWHM), defined at *m/z* 400 and five MS2 scan events (with a resolving power of 35,000 FWHM, defined at *m/z* 200). In the MS2 events, the precursor ion ranges, *m/z* 95 to 205, 195 to 305, 295 to 405, 395 to 505, and 495 to 1,005 were selected consecutively, by setting an inclusion list containing the following precursor ions: *m/z* 150, 250, 350, and 450 (with an isolation window of 110 *m/z*) and *m/z* 750 (with an isolation window of 550 *m/z*). The range of precursors with higher masses was set larger than the other ones because fewer resistance related (RR) metabolites were expected to fall within in this range. A stepped collision energy at 30 and 80 normalized collision energy, automatic gain control target 1 × 106, and maximum injection time of 200 ms were applied in all MS2 events. The system was controlled by the Xcalibur (version 3.1), Chromeleon MS Link 6.8, and Q-Exactive Tune 2.8 software package.

### Step 4: Data Processing (MZmine)

The obtained raw LC-HRMS data files were processed using the MZmine 2.5 software (Pluskal et al., 2010). The software can be downloaded for free at the following link: http://mzmine.github.io/. Continuous data, acquired in positive mode and negative mode, were processed separately. Data processing (feature extraction) with MZmine comprised the following steps: import data, MS peak detection, chromatogram building, chromatogram deconvolution, isotope grouping, first alignment (samples belonging to the same sample group were merged into a single peak list, so four peak lists were obtained for each sample group), MS row filtering, gap filling, second alignment (previous peak lists were aligned in one peak list), manual inspection, and principal component analysis (PCA). In these steps, the retention time tolerance was set after checking the maximum retention time of the internal standard ([^13^C_34_]–fumonisin B_1_) between all samples.

MZmine modules involved in data processing and relevant settings are summarized in Table 1.

**TABLE 1:** MZmine modules involved in data processing and relevant settings.

Menu	Module	Item and settings
Raw data methods	Raw data import	
	Filtering	Crop-filtered (retention time: 4–41 min, polarity: positive, spectrum type: profile, *m/z*: autorange)
	Peak detection (mass detection)	Mass detector: “exact mass”, noise: 1 × 10^5^, MS level:1
	Peak detection (FTMS shoulder peak filter)	Mass resolution: 70,000, peak module function: Lorentzian extended
	Peak detection (chromatogram building)	Minimum time span: 0.3 min, minimum height: 1 × 10^5^, *m/z* tolerance: 0.001 *m/z* or 5 ppm
Peak list methods	Peak detection (chromatogram deconvolution)	Algorithm: local minimum search (chromatographic threshold: 40%, search minimum in retention time range: 0.3 min, minimum relative height: 5%, minimum absolute height: 1.0 × 10^5^, minimum ratio of peak top/edge: 3 and peak duration range: 0.3–1.5 min
	Isotopes (isotopic peak grouper)	(*m/z* tolerance: 0.001 *m/z* or 5.0 ppm, retention time tolerance: 0.3 min absolute, maximum charge: 2, and representative isotope: most intense).
	Alignment	Join alignment: *m/z* tolerance: 0.001 *m/z* or 5.0 ppm, weight for *m/z*: 50, retention time tolerance 0.3 absolute, weight for RT: 50
	Gap filling	Same retention time and *m/z* range gap filler, *m/z* tolerance:0.001 *m/z* or 5.0 ppm
	Filtering	Peak list row filter (minimum peaks in a row: three, keep rows that match all criteria)
	Alignment	Join alignment: *m/z* tolerance: 0.001 *m/z* or 5.0 ppm, weight for *m/z*: 50, retention time tolerance 0.3 absolute, weight for retention time: 50
Project	Set sample parameters	Add experimental parameter (name: type sample, set of value, values “C_5_3,” “I_5_3,” “C_1484,” “I_1484”)
Peak list methods	Data analysis	Principal component analysis, data files: select all, peaks: select all, coloring style: coloring by parameter type

### Step 5: Custom Database Search (MZmine)

The module “custom database search” is included in MZmine. This module allows identifying peaks by consulting a database created by the user. In this study, a *Fusarium*-specific database was drafted in an Excel spreadsheet containing the following information for each compound, arranged in columns: ID, *m/z* (exact mass of [M+H]^+^ for ESI positive mode and [M-H]^–^ for ESI negative mode), molecular formula, chemical name, and retention time (this parameter was set equal to 0 if retention time was unknown). The database was saved into .csv format. After database matching (Table 2), an adduct search and a complex search were performed. Finally, the peak list was directly exported from MZmine in .csv (comma separated values) format compatible with MetaboAnalyst 4.0 web server^2^ (Xia et al., 2015).

**TABLE 2:** Custom database search (MzMine) module and settings.

Menu	Module	Setting
Peak list methods	Identification—custom database search	Database file: *Fusarium* DB, field separator:, filed order: ID, identity, formula, *m/z*, retention time (min), *m/z* tolerance:0.001 *m/z* or 5 ppm, retention time tolerance: 0.3 absolute
Peak list methods	Identification—adduct search	Adduct: Na-H, NH4, RT tolerance: 0.3 absolute (min), *m/z* tolerance: 0.001 *m/z* or 5.0 ppm, max relative adduct peak height: 30%
Peak list methods	Identification—complex search	Ionization method: [M + H]^+^ for ESI positive mode, [M - H]^–^ for ESI negative mode, retention time tolerance: 0.3 absolute (min), *m/z* tolerance: 0.001 *m/z* or 5.0 ppm, and with maximum complex peak height of 50%

### Step 6: Statistical Analysis (MetaboAnalyst)

The overall data set (.csv format) was opened in Excel, and it was split into three matrices (data sets) containing features related to the following sample groups: (a) *Fusarium*-inoculated RIL 14_84 versus mock-inoculated RIL 14_84, (b) *Fusarium-*inoculated RIL 5_3 versus mock-inoculated RIL 5_3, and (c) mock-inoculated RIL 5_3 versus mock-inoculated RIL 14_84. Each data set was saved into .csv format and subjected to univariate analysis in the MetaboAnalyst 4.0 web server (see text footnote 2) (Xia et al., 2015) by selecting the “statistical analysis” module (Table 3).

**TABLE 3:** MetaboAnalyst statistical analysis module: items and settings.

Item	Settings
Data type	Peak intensity table, format: sample in column (unpaired),
Missing value	Estimate the remaining missing values replace by a small value (half of the minimum positive value in the original data)
Data filtering	Interquartile range
Normalization	Sample normalization: none, data transformation: none, data scaling: Pareto scaling
Univariate analysis: volcano plot	*x* axis—fold change threshold: 2.0 *y* axis—*P* value threshold: 0.01 FDR-adjusted

Volcano plot analysis displays the fold change (FC) differences and the statistical significance for each variable (*p* value). For each feature, the FC is computed as the ratio between peak areas (mean value of the replicates) of the two compared sample groups. The log of the FC is plotted on the *x* axis so that changes in both directions (up and down) appear equidistant from the center. The *y* axis displays the negative log of the *p* value from the two-sample *t* test. In this study, metabolite features with a *p* value [corrected by false discovery rate (FDR)] less than 0.01 and FC greater than two were considered both statistically significant and biologically important. False discovery rate (Yoav and Yosef, 1995) was used for controlling the multiple testing problem, that is, the accumulation of false-positive results (type I error) when a confidence-based statistical test (the *t* test in the present case) is applied in parallel across multiple features.

Once statistical analysis was performed, results for each comparison were exported as graph (volcano plot) and .csv file. The latter contained for each significant feature values of “FC” “log2(FC)” “p.adjusted,” “-log10(p),” where adjusted is *p* value FDR corrected.

### Step 7: Identity Confirmation/Compound Identification

Identity of putative metabolites was confirmed on the basis of MS/MS fragment ions (measured in so-called DIA mode) followed by database search on Mass Bank^3^, METLIN^4^, and MzCloud^5^. In METLIN, the Advanced Search tool was selected to search for MS/MS fragments of metabolites based on different parameters such as name, simplified molecular-input line-entry system (SMILES), Kyoto Encyclopedia of Genes and Genomes (KEGG) number. Accurate masses of the fragments obtained in the same ionization mode were matched with the database by setting a tolerance of 5 ppm. Compound names were also inserted in the search tool of MzCloud to look for MS/MS fragments.

### Step 8: Biochemical Interpretation

A pathway analysis was performed to better elucidate the function of the altered metabolites by using MetaboAnalyst 4.0, via KEGG pathway database^6^, compared with *Oryza sativa* ssp. japonica (Rice Annotation Project Data Base^7^) pathway library (Chong et al., 2019).

## Results and Discussion

**FIGURE 2:**
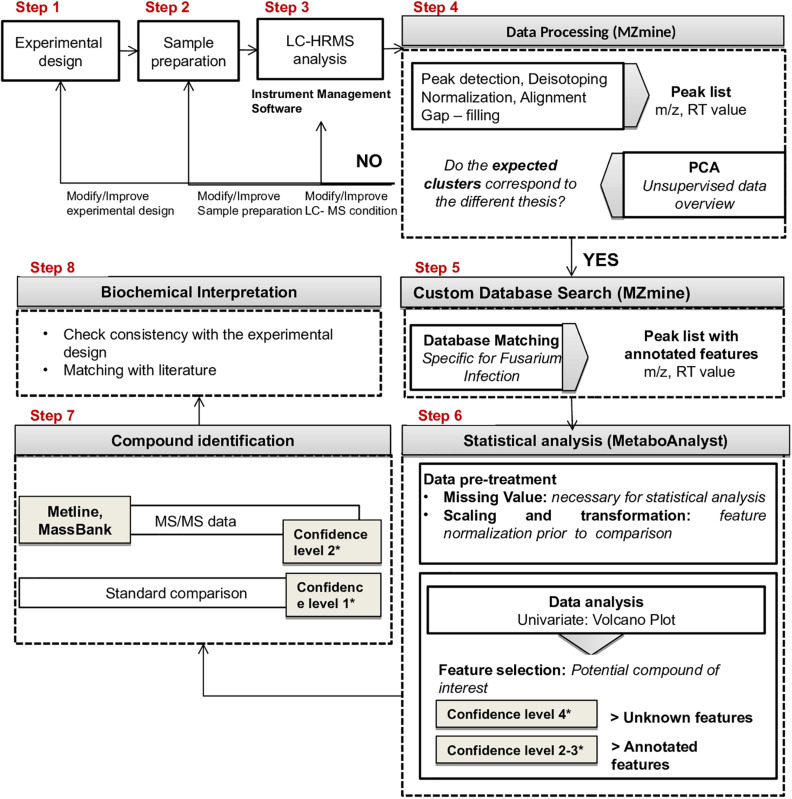
Proposed workflow for LC-HRMS metabolomics studies.

The proposed workflow included the following steps (Figure 2): (1) experimental design; (2) sample preparation; (3) LC-HRMS analysis; (4) data processing (MZmine 2.35^8^); (5) custom database search against a *Fusarium* specific database (MZmine2.35, see text footnote 8); (6) statistical analysis (Metaboanalyst 4.0, see text footnote 2); (7) compound identification; (8) biochemical interpretation.

Compounds were classified according to confidence levels as defined by the Compound Identification Workgroup of the Metabolomic Society (Sumner et al., 2007; Blaženovic et al., 2018), which are summarized in Table 4 together with relevant minimum data requirements. It is worth mentioning that open-source database and tools used in the workflow are frequently updated; this could slightly affect data reproducibility.

**TABLE 4:** Description and minimum data requirements for confidence levels of compound identification (redrafted from Blaženovic et al., 2018).

Confidence level	Description	Minimum data requirements
Level 0	Unambiguous 3D structure: isolated pure compound, including full stereochemistry	Following natural product guidelines, determination of 3D structure
Level 1	Confident 2D structure: uses reference standard match or full 2D structure elucidation	At least two orthogonal techniques defining 2D structure confidently, such as MS/MS and RT or CCS
Level 2	Probable structure: matched to literature data or databases by diagnostic evidence	At least two orthogonal pieces of information, including evidence that excludes all other candidates
Level 3	Possible structure or class: most likely structure, isomers possible, substance class or substructure match	One or several candidates possible, requires at least one piece of information supporting the proposed candidate
Level 4	Unknown feature of interest	Presence in sample

### Experimental Design, Sample Preparation, and LC-HRMS Analysis

**FIGURE 3:**
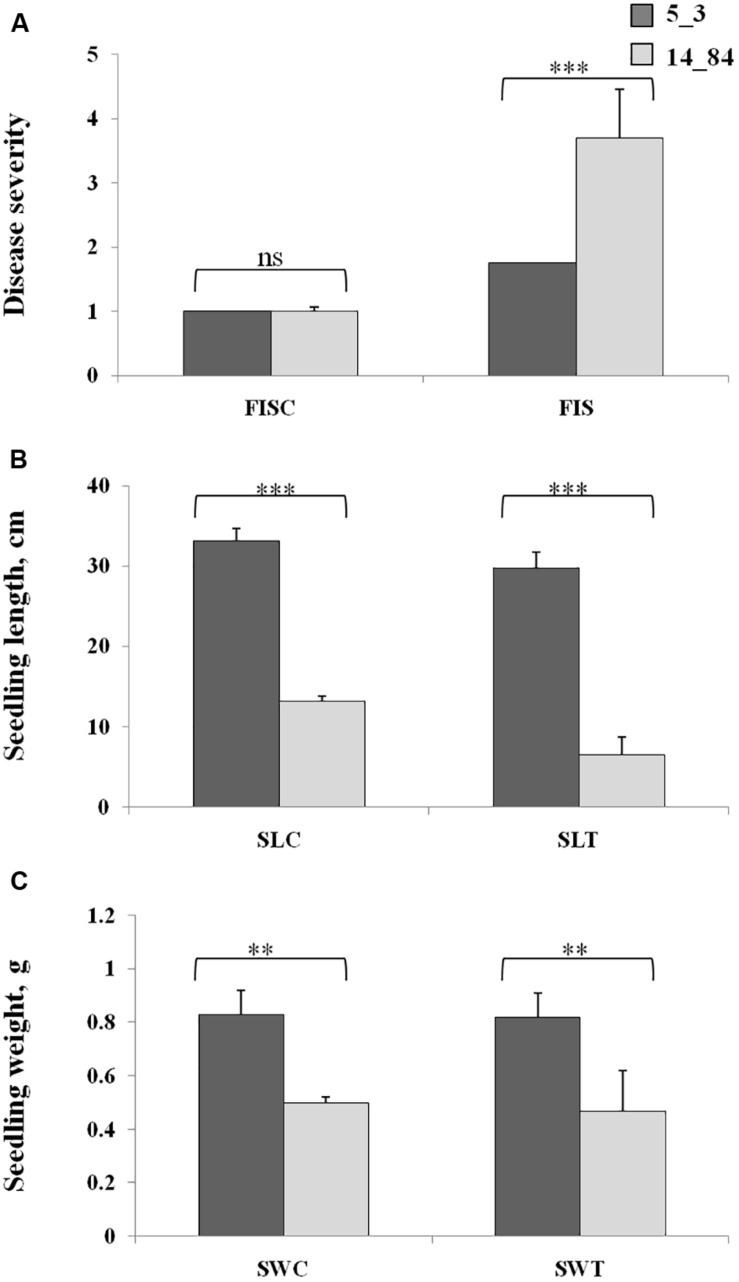
Phenotypic values of *Fusarium* infection of seedlings **(A)**, seedling length **(B)**, and seedling weight **(C)** in the control rolled towel assays (RTAs), named as FISC, SLC, and SWC, and treated RTAs, named as FIST, SLT, and SWT, in the recombinant inbred lines 5_3 (dark gray) and 14_84 (light gray). Asterisks (*) indicate significant differences between 5_3 and 14_84 control (mock inoculation) and means treated (*Fusarium* inoculated) within the same trait, according to two-way analysis of variance (**P ≤ 0.01; ***P ≤ 0.001; ns, not significant).

Workflow application started by planning a suitable experimental design to investigate the metabolic response of maize induced by *F. verticillioides* infection (Step 1). To verify the suitability of the maize RILs selected for the case-control study, their phenotypes were characterized with respect to disease severity after *F. verticillioides* infection of seedlings (FIS) by RTA screening. Rolled towel assay methodology was chosen instead of field experiments to minimize noncontrolled biological variations and environmental effects to be confident that the observations were due only to the investigated biological variation (i.e., infection by *Fusarium*). The RTAs resulted in minor symptoms of FIS for RIL 5_3 with respect to 14_84 at 7 days after inoculation (Figure 3). Furthermore, higher SL and weight values were measured for the RIL 5_3, supporting the better performance of this line.

For metabolite extraction and characterization, the experimental design has foreseen the comparison of the following sample groups (Figure 1): control (mock inoculation) versus *Fusarium-*inoculated kernels for both RILs 5_3 and 14_84 to have information on metabolites generated after the fungal infection (responsive metabolites) and then control (mock RIL 5_3) versus control (mock RIL14_84) to obtain information on differences in constitutive metabolites potentially related to defense mechanism. It is worth to point out that, for a more complex study design (e.g., multiple time point and treatment group), sample size calculation and power analysis should be considered for inclusion in the workflow. This step can be performed by MetaboAnalyst.

The need for an unselective sample extraction procedure is dictated by the need to analyze as wide a range of metabolites as possible (Vuckovic, 2012). Therefore, to maximize the extractable metabolic information (Step 2), a protocol involving two sequential extractions with methanol and acidified aqueous acetonitrile 84:16 (vol/vol) was applied. The two-step extraction procedure was preferred to a single extraction to further increase recoveries of less polar compounds (Ciasca et al., 2018). The addition of acetic acid was used to improve recoveries of FBs, which were key fungal metabolites in this study (Sulyok et al., 2006).

To ensure the reliability of the data generated by LC-HRMS (Step 3), three issues were considered: (i) use of internal standard, (ii) setting a broad scope acquisition mode for untargeted analysis, and (iii) use of an appropriate analysis sequence.

In this study, isotopically labeled fumonisin B_1_ was added as internal standard to the final test sample prior to LC-HRMS analysis. Only one substance was used as internal standard to not introduce further complexity in mass spectra, as recommended by De Vos et al. (2007). Although the internal standard was not representative for each class of samples analyzed, it could be considered a guarantee of system stability. To check for system stability, the sample set was injected twice, and the coefficient of variation of internal standard area over the whole injection sequence (CVa) was calculated. The system was considered stable if CVa was less than 20%. In this study, CVa resulted less than 15%. In addition, the maximum variation of internal standard retention time ([^13^C_34_]–fumonisin B_1_) was applied as filter criterion (tolerance value) during retention time alignment in data processing (Section “Step 4: Data Processing (MZmine)”). An alternative approach to check and correct signal drift based on the use of pooled QC samples (mix of all samples) has been proposed (Dunn et al., 2012; Broadhurst et al., 2018). This approach, together with multiple internal standard, is recommended particularly for studies considering a large number of samples.

To set up a broad-scope acquisition method, the full-scan/vDia acquisition modes were used (see section “Step 3: LC-HRMS Analysis”). The alternation of two scan events with and without fragmentation allowed obtaining product ions for identity confirmation of the detected metabolites. Moreover, two separate runs were acquired in positive and negative ion modes, respectively.

The last issue to be considered was the analysis sequence. Samples were injected in random order to avoid any possible artificial sample aggregation due to the analytical drift. A blank injection of 100% methanol was run at the beginning and at the end of the sample set to check for carryover effects. The described approach was developed on an HRMS instrument based on Orbitrap^TM^ analyzer. However, full scan and vDIA acquisition modes can be also implemented on quadrupole time-of-flight mass spectrometers, and similar information on the metabolite structure can be expected, provided that same experimental conditions are applied.

### *m/z* Data Processing: MZmine

**FIGURE 4:**
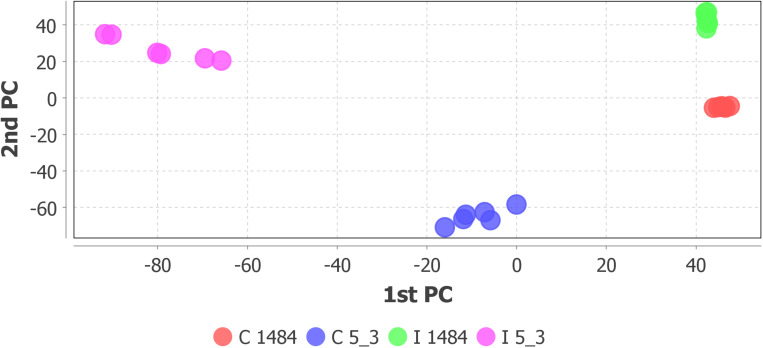
Score scatter plot of principal component analysis relative to peak list from LC-HRMS chromatograms (positive ionization) of the sample set: RIL5_3 Fusarium inoculated (pink dots), RIL5_3 control (blue dots), RIL14_84 Fusarium inoculated (green dots), RIL14_84 control (red dots).

Step 4 of the workflow foresees features extraction (monoisotopic *m/z*, charge, retention time, peak width, and peak area) from full scan chromatograms acquired in positive and negative ion mode. Liquid chromatography–HRMS raw data processing was performed using MZmine version 2.35. The optimized MZmine parameters for features extraction are reported in Tables 1, 2. Parameters for filtering, peak detection, and gap filling were set on the basis of the applied LC-HRMS conditions (f.i. gradient elution, mass resolution, observed peak width, and relative intensity). A peak list of 6,363 features from positive ionization acquisitions and 3,736 features from negative ionization acquisitions was obtained. Before proceeding to next steps (custom database search and statistical analysis, Figure 2), PCA was performed to check the overall quality of the analytical system and method performance and to visually inspect for samples out of clusters. Figure 4 depicts the score plot of PCA relevant to the peak list from LC-HRMS chromatograms acquired in positive ionization mode. The different colors indicate data from samples from the different thesis (i.e., the RIL5_3 *Fusarium* inoculated, RIL 5_3 control, RIL14_84 *Fusarium* inoculated, RIL 14_84 control) for a better visualization in the score plot.

The score scatter plot (Figure 4) shows a very clear separation between the two RILs (5_3 and 14_84) and the treatments (mock and *Fusarium* inoculation). The first principal component (1st PC) explains differences between line 5_3 (negatively correlated) and line 14_84 (positively correlated), whereas the second principal component (2nd PC), explains differences between control samples (negative correlated) and inoculated samples (positive correlated). Because visual inspection of PCA showed a sample clustering matching with the four thesis as defined in the experimental design (Figure 1) and given the absence of samples out of clusters, indicating the suitability of the experimental design as well as of the feature extraction settings, it was possible to proceed to the next step. When PCA shows undefined clusters, this is an objective indication that improvements of LC-HMRS conditions and/or sample preparation and/or the experimental design are needed (Figure 2).

### Custom Database Search (MZmine)

In Step 5, all the extracted features (peak list) were matched against a *Fusarium*-specific database using the “custom database search” tool of MZmine (section “Materials and Methods”). The database used in this study included a total of 764 metabolites known to be involved in *Fusarium* infection of cereals. In particular, the database included metabolites identified in previously published studies dealing with *Fusarium* infection in grains and all metabolites listed in the “Wheat *Fusarium* Head Blight Disease” (MWFD) database^9^. The whole database is provided in Supplementary Table S1. Where available, the use of a specific database offers advantages in terms of saving time and biological meaning of the annotation, because it returns only compounds related to the studied event. As additional information, the “adduct search” tool of MZmine was used to identify for each ion feature the main adducts that could be formed in electrospray ion source.

Finally, the peak list returned by the custom database search was exported into a .csv file suitable to be directly uploaded into the Statistical Analysis module of the MetaboAnalyst service (Step 6).

### Statistical Analysis (MetaboAnalyst)

**FIGURE 5:**
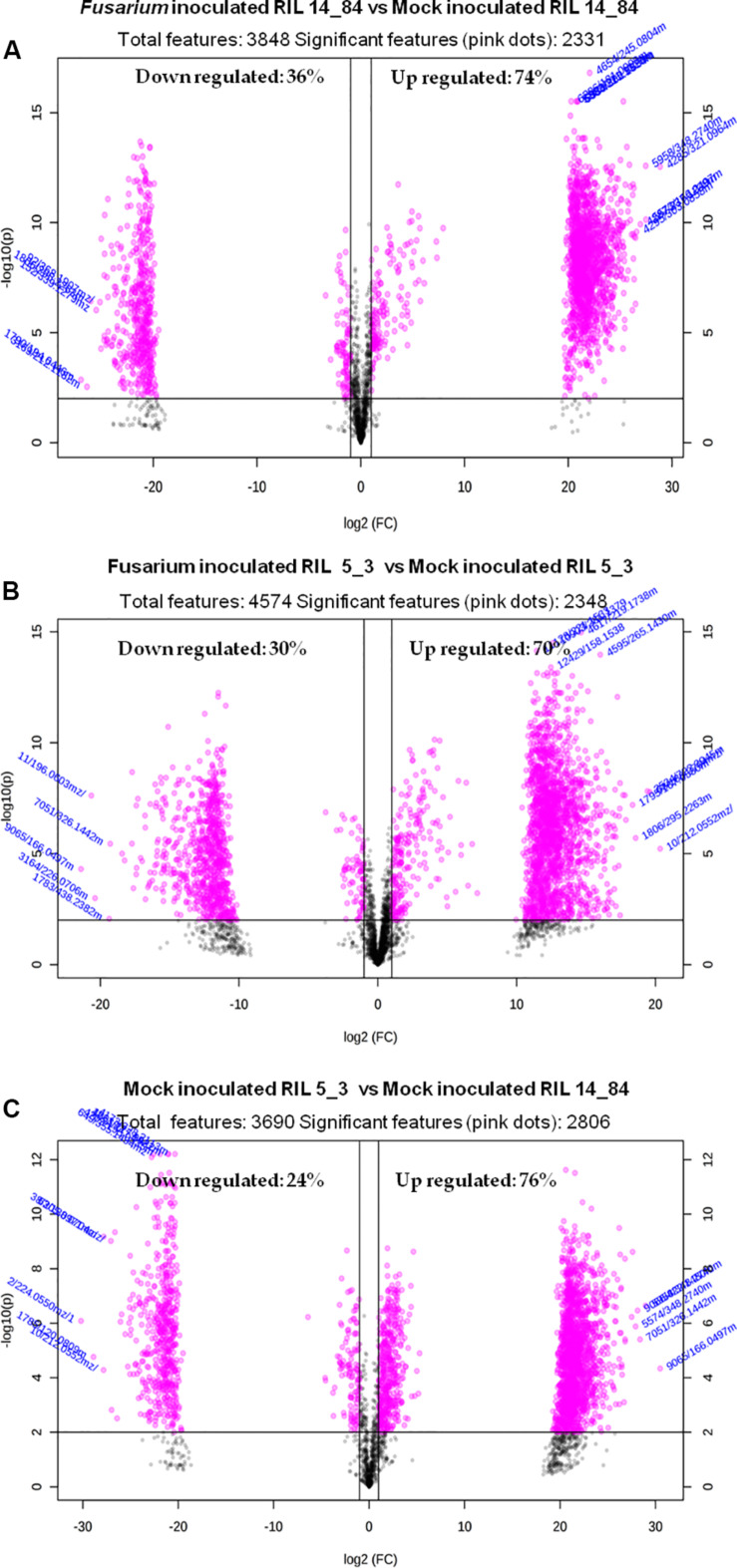
Volcano plots relevant to the comparison of the extracted features of **(A)** *Fusarium*-inoculated RIL 14_84 versus mock-inoculated RIL 14_84, **(B)** *Fusarium-*inoculated RIL_5_3 versus mock-inoculated RIL 5_3, **(C)** mock-inoculated RIL 5_3 versus mock-inoculated RIL 14_84. Gray dots indicate features not significantly different between the compared sample groups. Pink dots indicate features significantly different between the compared sample groups (absolute value of FC > 2, *P* < 0.01).

Because of the costs and efforts required to identify unknown metabolites, a preliminary selection of the features (Step 6) was applied prior to further identification and confirmation steps. The applied criterion was to select only features showing statistically significant differences after comparison between the sample groups (thesis) as defined in the experimental design (Figure 1). For this purpose the volcano plot was used to identify the largest and most significantly changing features in (a) *Fusarium*-inoculated RIL 14_84 versus mock-inoculated RIL 14_84, (b) *Fusarium-*inoculated RIL_5_3 versus mock-inoculated RIL 5_3, (c) mock-inoculated RIL 5_3 versus mock-inoculated RIL 14_84. Volcano plot analysis reveals metabolite features that are up-regulated and down-regulated by *p* value and FC analysis (Figure 5). Black lines in the plots indicate the *p* value threshold (0.01, horizontal line) and the FC threshold (2, vertical line). The upper quadrants contain the significant features (up-regulated features on the left, down-regulated ones on the right, Figure 5). The comparisons of features detected in *F. verticillioides*–infected and mock-inoculated maize kernels (controls) revealed that after inoculation there was a major number of up-regulated features with respect to down-regulated ones in both inbred lines (70 and 74% for the 5_3 and 14_84 genotype, respectively). Moreover, when comparing mock samples, RIL 5_3 exhibited a higher percentage of up regulated features with respect to RIL 14_84.

For each profile comparison (volcano plot), MetaboAnalyst also provides a .csv file containing the list of significant features characterized by monoisotopic mass *m/z*, retention time, and compound name, with the respective values of *p* and FC. Unidentified features are termed as unknown compounds. Significant features listed in the .csv file were then subjected to inspection of mass fragmentation pattern and comparison with reference standards (Step 7) to achieve a confidence level in identification between 2 and 4 (Table 4).

### Identify Confirmation/Compound Identification

In Step 7, identity confirmation of putative metabolites (features listed in the .csv file from Step 6) was performed by matching the detected fragments, molecular ion and retention time with molecular ion, fragmentation pattern in METLIN (see text footnote 4), and Mass Bank^10^ databases. Table 5 lists compounds showing an MS/MS fragmentation pattern matching fully (identification level 2) or partially (identification level 3) with consulted MS/MS databases. Reproducibility of MS/MS spectra is an issue of utmost importance for compound identification. Whereas the type of generated fragments is more related to the chemical structure of metabolite itself and is expected to be reproducible in different mass analyzer, some complication in interpreting MS/MS spectra could be caused by changes in product ions ratios. Therefore, it is important to compare MS/MS spectra obtained in double stage mass analyzers, that is, able to isolate the precursor ion and to fragment it in controlled collision conditions (collision energy and gas flow), rather than using fragmentation products generated in the ion source.

**TABLE 5:** List of significant metabolites (*P* ≤ 0.01 and relative changes FC > | 2|) classified as *Fusarium verticillioides* (Fv)–responsive maize metabolites specific to RIL 14_84 (derived from volcano plots *Fusarium*-inoculated RIL 14_84 vs. mock-inoculated RIL 14_84), specific to RIL5_3 (*Fusarium-*inoculated RIL_5_3 vs. mock-inoculated RIL 5_3), common to both genotypes, and constitutive (mock-inoculated RIL 5_3 vs. mock-inoculated RIL 14_84).

Putative name	IUPAC name	Rt (min)	Adduct M+H/M+H-H2O*	Fragment ions (*m/z*)	Class	Assigned status
L-Tryptophan	5,7-Dihydroxy-2-(4hydroxyphenyl)-4*H*-chromen-4-one	7.2	205.09715	188.0704, 146.0599, 144.0807, 159.0916, 118.06541	AA	Constitutive 14_84
Tyrosine	(2*S*)-2-amino-3-(4-hydroxyphenyl)propanoic acid	14.4	182.08117	136.0757, 165.0547, 123.0440	AA	Constitutive 5_3; Common
Apigenin	5,7-Dihydroxy-2-(4-hydroxyphenyl)-4*H*-chromen-4-one	25.7	271.0601	153.0182, 119.0491	PP FL: flavone	Fv responsive 14_84
*N*-coumaroylserotonin	2E)-*N*-[2-(5-Hydroxy-1*H*-indol-3-yl)ethyl]-3-(4-hydroxyphenyl) acrylamide	24.1	323.1390	nd	PP not FL	Fv responsive 14_84
*N*-feruloylserotonin	(2E)-*N*-[2-(5-Hydroxy-1*H*-indol-3-yl)ethyl]-3-(4-hydroxy-3-methoxyphenyl) acrylamide	24.8	353.1496	nd	PP not FL	Common
Feruloyltryptamine	(*E*)-3-(4-hydroxy-3-methoxyphenyl)-*N*-[2-(1*H*-indol-3-yl)ethyl]prop-2-enamide	30.7	307.1441	119.0943	PP not FL	Common
Fumonisin B1	2-[2-[19-Amino-6-(3,4-dicarboxybutanoyloxy)-11,16,18-trihydroxy-5,9-dimethylicosan-7-yl]oxy-2-oxoethyl]butanedioic acid	31.1	722.3939	370.3316, 352.3210, 334.3104, 316.2998	Mycotoxins	Common
Fumonisin B2	(2*R*)-2-[2-[(5*R*,6*R*,7*S*,9*S*,16*R*,18*S*,19*S*)-19-amino-6-[(3*R*)-3,4-dicarboxybutanoyl]oxy-16,18-dihydroxy-5,9-dimethylicosan-7-yl]oxy-2-oxoethyl]butanedioic acid	36.0	706.3995	688.3903, 530.3687, 512.3582, 354.3366, 336.3261	Mycotoxins	Common
Fumonisin B3	2-[2-[(5*R*,6*R*,7*S*,9*S*,11*R*,18*R*,19*S*)-19-amino-6-(3,4-dicarboxybutanoyloxy)-11,18-dihydroxy-5,9-dimethylicosan-7-yl]oxy-2-oxoethyl]butanedioic acid	33.9	706.3995	688.3903, 30.3687, 512.3582, 354.3366,336.3261	Mycotoxins	Common
Fusarin C	Methyl (2*E*,3*E*,5*E*,7*E*,9*E*)-2-ethylidene-11-[(1*R*,4*S*,5*R*)-4-hydroxy-4-(2-hydroxyethyl)-2-oxo-6-oxa-3-azabicyclo[3.1.0]hexan-1-yl]-4,6,10-trimethyl-11-oxoundeca-3,5,7,9-tetraenoate	35.8	432.2017	273.14851, 213.1271, 111.0446	Mycotoxins	Common
Kaempferol-3-*O*-glucoside-7-*O*-rhamnosideisomer	5-Hydroxy-2-(4-hydroxyphenyl)-3-[(2*S*,5*S*)-3,4,5-trihydroxy-6-(hydroxymethyl)oxan-2-yl]oxy-7-[(2*S*,4*S*,5*R*)-3,4,5-trihydroxy-6-methyloxan-2-yl]oxychromen-4-one	18.7	595.1657	449.1078, 287.0550, 121.0284	PP FL: flavanols	Constitutive 5_3
Kaempferol-3-*O*-glucoside7-*O*-rhamnosideisomer		21.7	595.1657	nd	PP FL: flavanols	Constitutive 5_3
Kaempferol-3-*O*-glucoside7-*O*-rhamnosideisomer		20.9	595.1657	nd	PP FL: flavanols	Constitutive 5_3
Narigenin	5,7-Dihydroxy-2-(4-hydroxyphenyl)chroman-4-one	29.2	273.0753	153.0186,119.0491	PP FL: flavanone	Fv responsive 14_84
Narigeninchalchone	(E)-3-(4-hydroxyphenyl)-1-(2,4,6-trihydroxyphenyl)prop-2-en-1-one	30.6	273.0753	153.0186, 147.0441,119.0491	PP FL: flavanone	Common
Tetrahydroxy- (methylsuccinoyl)flavon	2-Methyl-4-oxo-4-[3,5,7-trihydroxy-2-(4-hydroxyphenyl)-4-oxochromen-8-yl]butanoic acid	40.5	383.0755*	nd	flavonoids	Common

*AA, amino acids; PP FL, phenylpropanoids flavonoids; PP not FL, phenylpropanoids not flavonoids; FA, fatty acids; ns, not significant (FC > 2, P > 0.01); nd, fragments not detected. MS/MS fragments in bold characters indicate the actual match of the fragments in the database.*

### Biochemical Interpretation

For Step 8 (biochemical interpretation), the Pathway Analysis was performed on significantly altered metabolites by using *O. sativa* japonica to associate the biological functions of identified metabolites to different pathways (Table 5 and Supplementary Table S2). Five of 14 metabolites listed in Table 5 were assigned to 10 different metabolic pathways, and the pathways flavonoid biosynthesis; phenylalanine, tyrosine, and tryptophan biosynthesis; and aminoacyl-tRNA biosynthesis showed a higher number of metabolites (Supplementary Table S2). The latter ones included the two amino acids L-tryptophan and tyrosine, whereas the three compounds apigenin, naringenin, and naringenin chalchone belonged to the flavonoid biosynthesis pathway (Supplementary Table S2).

The detected L-tryptophan and tyrosine, together with phenylalanine, are key amino acids for the synthesis of compounds of the secondary metabolism, and their biosynthesis is issued from the shikimate pathway. L-Tryptophan was observed at constitutive level in line 14_84 with high level of infection (log_2_FC = 27.8 for ratio mock-inoculated RIL 5_3 versus mock-inoculated RIL 14_84). On the other hand, tyrosine was found at higher levels in the 5_3 mock-treated samples (log_2_FC = 2.4) and induced after *F. verticillioides* inoculation in both genotypes (log_2_FC = 14.5 vs. 21.1 for the 5_3 and 14_84 genotypes, respectively).

Several flavonoids accumulated after mock and fungal treatment. Notably, the metabolite kaempferol-3-*O*-glucoside 7-*O*-rhamnoside displayed elevated levels only in the mock 5_3 samples with log_2_FC values of approximately 22. Additionally, the flavone apigenin and the flavanone naringenin were significantly modulated after *F. verticillioides* inoculation. In previous works, naringenin was described as a potential biomarker for resistance to *Fusarium* head blight in some wheat and barley cultivars (Kumaraswamy et al., 2011; Gunnaiah et al., 2012) and was reported as an efficient inhibitor of *in vitro* growth of *F. graminearum* (Bollina et al., 2010). The flavonoid tetrahydroxy-(methylsuccinoyl)flavone was detected in both lines and showed a common enhancement after fungal infection (log_2_FC = 6.11 vs. 5.2 for 14_84 and 5_3 genotypes, respectively). This compound was found affecting fumonisin accumulation in maize, because it was abundant in highly contaminated maize samples (Righetti et al., 2019). The identification of almost 100 putative flavonoids playing a part in the chemical repository of wheat and barley against *F. graminearum* was described by Gauthier et al. (2015). Furthermore, findings from Venturini et al. (2015) corroborated the involvement of flavonoids in the resistance of maize to *Fusarium* ear rot and fumonisin accumulation contributing to hardening of kernel pericarp, supporting the central role of this class of metabolites in maize defense responses.

In addition to the importance of flavonoids in the fight against fungal pathogens, metabolic profiling of 5_3 and 14_84 genotypes following fungal treatment also revealed the *N*-hydroxynnamic acid amides (HCAAs) coumaroylserotonin, feruloylserotonin, and feruloyltryptamine. *N*-hydroxynnamic acid amides are the results of the condensation of hydroxycinnamoyl-coA thioesters and aromatic amines, as tryptamine and serotonin both derive from the identified tryptophan. N-hydroxynnamic acid amides can be regarded as metabolic intermediates and accumulate in the cell wall acting as physical barrier against pathogens. Moreover, they reduce the plant digestibility, inhibit fungal hyphae growth, and are induced by physical injury, pathogen infection, and elicitor treatment (Balmer et al., 2013; Macoy et al., 2015). Serotonin and its HCAAs, p-coumaroylserotonin and feruloylserotonin, were also accumulated in *Bipolaris oryzae* infected leaves of rice (Ishihara et al., 2008) and in resistant wheat cultivars after *F. graminearum* infection (Gunnaiah et al., 2012). Interestingly, in this study, the feruloylserotonin and feruloyltryptamine metabolites were observed in both genotypes after *F. verticillioides* infection, whereas the coumaroylserotonin showed enhanced levels specifically in the 14_84 genotype (log_2_FC = 23.8).

The seeds of the two maize selected lines were also analyzed for fumonisin and fusarin content (Table 5). After *F. verticillioides* inoculation, both genotypes exhibited the presence of mycotoxins, with slightly higher levels of fumonisin B_1_ in the 5_3 treated samples (log_2_FC = 15.7 vs. 6.1 for the 5_3 and 14_84 genotypes, respectively). Despite the line 5_3 displayed lower severity of the disease compared to the 14_84 genotype, the fungus was able to synthesize toxic metabolites inside kernel tissues. A low relation between disease intensity and levels of fumonisins could be an inherent factor of this pathosystem, a hypothesis also reinforced by other studies. Afolabi et al. (2007) suggested that genetic factors that affect grain infection may act independently of those affecting fumonisin production. Moreover, the quantitative genetic nature of these two traits could partly explain the presence of mycotoxins even in case of such asymptomatic infection (Munkvold and Desjardins, 1997; Saunders et al., 2001; Sánchez-Rangel and SanJuan-Badillo, 2005; Picot et al., 2010; Rosa Junior et al., 2019).

In this regard, much effort should be addressed at selecting genetic material resistant to both characteristics, because there is not always a relationship between fungal infection and fumonisin production.

### Verification

To obtain preliminary information on the reproducibility of the developed workflow, the procedure was applied to a further sample set. Mock (control)– and *F. verticillioides–*inoculated samples were prepared for a maize RIL (18_27) characterized by a high level of infection (severity value approximately 3.9). Samples were processed according to the workflow in Figure 2.

After extraction and LC-HRMS analysis, data processing by MZmine returned a score plot of PCA with two sample clusters relating to *Fusarium-*inoculated samples and mock-inoculated samples. In agreement with the first case study, mock-inoculated samples were negatively correlated, whereas *Fusarium-*inoculated ones showed a positive correlation along 1st PC.

Volcano plot analysis revealed a slightly higher number of up-regulated features (57%) with respect to down-regulated (43%) ones. Results were quite similar to those obtained from the comparison *Fusarium-*inoculated versus control for RIL14_84 showing very close disease severity values (3.7 vs. 3.9 for RIL 14_84 and RIL 18_27, respectively).

Identity confirmation of putative metabolites among features listed in the .csv file after profile comparison (volcano plot) analysis led to the identification of mycotoxins B_1_ (log_2_FC:15.7), fumonisin B_2_ (log_2_FC:13.6), fumonisin B_3_ (log_2_FC:13.7), and fusarin C (log_2_FC:14.8). Moreover, there was an increase of apigenin (log_2_FC:2.6), feruloylserotonin (log_2_FC:4.5), and p-coumaroylserotonin (log_2_FC: 4.7) after inoculation with *F. verticillioides*. Fold change values were higher than those obtained for RIL14_84 (apigenin, log_2_FC:2.6 vs. 24.2, feruloylserotonin, log_2_FC: 4.5 vs. 22.8, p-coumaroylserotonin, log_2_FC: 4.7 vs. 23.8 for RIL 1,827 and 1,484, respectively).

Amino acids such as L-tryptophan (log_2_FC:-17.3) and L-phenilalanine (log_2_FC:-21.35) were more abundant in mock-inoculated compared with *Fusarium*-inoculated in RIL 18_27. L-Tryptophan was also found such as constitutive metabolite of line 14_84.

Therefore, coumaroylserotonin and apigenin were confirmed to be a *F*. *verticillioides*–responsive metabolite, whereas L-tryptophan was a putative constitutive metabolite being detected in two RILs showing high disease severity.

## Conclusion

In the present work, a workflow based on open-source and user-friendly tools for LC-HRMS plant metabolomics studies was presented. The workflow, covering all key steps from the experimental design to biochemical interpretation, allows identifying candidate metabolites in a single LC-MS analysis sequence.

Its applicability was evaluated through the study of metabolomics changes of two maize RILs with contrasting phenotypes with respect to disease severity after to *F. verticillioides* infection of seedlings. Constitutive metabolites and responsive metabolites belonging to different metabolic pathways were identified. In particular, kaempferol-3-*O*-glucoside7-*O*-rhamnoside and tyrosine were classified as constitutive metabolites of RIL 5_3 showing minor disease symptoms, whereas tryptophan was more abundant at constitutive level in RIL 14_84 with higher disease severity. Moreover, after fungal infection in RIL 14_84 was observed, an increment of apigenin, coumaroylserotonin, and naringenin. Finally, feruloyl-tryptamine, tryptophan, and several mycotoxins were detected such as responsive metabolites common to both lines.

These findings were confirmed in a verification study suggesting the suitability of the proposed workflow for future validation studies (a possible approach has been reported by Naz et al., 2014) including interlaboratory comparison. These data will enable to evaluate the applicability of the proposed workflow to larger studies.

The proposed workflow has been designed for the study of the metabolic profiles of plants. It describes and discusses each step, from sample preparation to data analysis and biochemical interpretation. Indeed, other tools have been described in the literature (Dunn et al., 2011, XCMS Online^11^, Galaxy^12^). However, the proposed work describes the process of adapting available tools to a specific control-case study, such as investigating on changes in plant metabolite profile after fungal infection. MetaboAnalyst was chosen among the available tools, because it offers the most complete support for statistical analysis, functional interpretation, and integration with other -omic data. Even though the MetaboAnalyst modules have not been fully exploited in this study, the perspective of integrating them in further steps of the workflow for future developments remains open. MZmine was used prior to MetaboAnalyst given its better performances in feature extraction from raw data.

## Data Availability Statement

All datasets generated for this study are included in the article/Supplementary Material.

## Author Contributions

BC, VL, and AL: conceptualization. BC and AL: formal analysis. BC, AL, VL, and MP: methodology. VL, AL, and AFL: supervision. BC and AL: writing—original draft. BC, VL, AL, MP, AM, and AFL: writing—review and editing.

## Conflict of Interest

The authors declare that the research was conducted in the absence of any commercial or financial relationships that could be construed as a potential conflict of interest.

## Footnotes

1 http://server.ispa.cnr.it/ITEM/Collection

2 http://www.metaboanalyst.ca

3 http://www.massbank.jp/?lang=en

4 http://metlin.scripps.edu

5 https://www.mzcloud.org/

6 http://www.genome.ad.jp/kegg/pathway.html

7 http://rapdb.dna.affrc.go.jp

8 http://mzmine.github.io/

9 https://bioinfo.nrc.ca/mwfd/index.php

10 www.massbank.jp

11 https://www.ncbi.nlm.nih.gov/pmc/articles/PMC3703953/

12 https://www.ncbi.nlm.nih.gov/pmc/articles/PMC5192046/
