# Supplementary material for: Application of an Integrated and Open Source Workflow for LC-HRMS Plant Metabolomics Studies. Case-Control Study: Metabolic Changes of Maize in Response to Fusarium verticillioides Infection
Source: Front Plant Sci. 2020 Jun 5;11:664. doi: 10.3389/fpls.2020.00664 (PMC7290002; doi:10.3389/fpls.2020.00664)
Supplement: Supplementary file 2 [file Data_Sheet_1.docx]

**Supplementary Table 2:** Pathway names, total metabolites involved in those pathways, metabolites significantly accumulated in the present study and classified as *Fusarium verticillioides* (FV)-responsive maize metabolites specific to RIL 14_84, specific to RIL5_3 (*Fusarium* inoculated RIL_5_3 vs mock inoculated RIL 5_3), common to both genotypes, and constitutive (mock inoculated RIL 5_3 vs mock inoculated RIL 14_84), and false discovery rate (FDR) identified by Pathway Analysis of MetaboAnalyst 4 using *Oryza sativa* japonica as the pathway library.

| **Pathway name** | **Total n°of metabolites** | **N° of metabolites involved in this study** | **Metabolites in involved in** | | | | **FDR** |
| --- | --- | --- | --- | --- | --- | --- | --- |
|  |  |  | *Fv responsive 14_84* | *Common* | *Constitutive 5_3* | *Constitutive 14_84* |  |
| Flavonoid biosynthesis | 47 | 3 | Naringenin, Apigenin | Naringeninchalchone |  |  | 0.032 |
| Phenylalanine, tyrosine and tryptophan biosynthesis | 22 | 2 |  | Tyrosine | Tyrosine | L-tryptophan | 0.108 |
| Aminoacyl-tRNA biosynthesis | 46 | 2 |  | Tyrosine | Tyrosine | L-tryptophan | 0.312 |
| Isoquinoline alkaloid biosynthesis | 6 | 1 |  | Tyrosine | Tyrosine |  | 0.504 |
| Flavone and flavonol biosynthesis | 12 | 1 | Apigenin |  |  |  | 0.799 |
| Tyrosine metabolism | 18 | 1 |  | Tyrosine | Tyrosine |  | 0.991 |
| Tryptophan metabolism | 23 | 1 |  |  |  | L-tryptophan | 1.0 |
| Glycine, serine and threonine metabolism | 33 | 1 |  |  |  | L-tryptophan | 1.0 |
| Ubiquinone and other terpenoid-quinone biosynthesis | 35 | 1 |  | Tyrosine | Tyrosine |  | 1.0 |
| Phenylpropanoid biosynthesis | 35 | 1 |  | Tyrosine | Tyrosine |  | 1.0 |
